# “THEY SHOULD PUT ALZHEIMER’S GROUPS IN THE CHURCHES”: A SYNTHESIS OF AFRICAN AMERICAN CAREGIVERS’ LIVED EXPERIENCES

**DOI:** 10.1093/geroni/igz038.3514

**Published:** 2019-11-08

**Authors:** Erin R Murphy, Destony Brooks, Julie Bryant, Noelle L Fields, Ling Xu

**Affiliations:** 1 University of Texas at Arlington, School of Social Work, Arlington, Texas, United States; 2 University of Texas at Arlington, Arlington, Texas, United States

## Abstract

Alzheimer’s disease and related dementias (ADRD) are challenging chronic health conditions that disproportionately impact African Americans. Caring for a family member with ADRD can be a taxing experience that impacts the mental, social, and physical realms of the caregiver’s life. Chronic fatigue and high levels of anxiety, depression, and agitation have all been associated with caregiving. The extant literature on caregivers is limited by being conducted primarily in settings with White participants, excluding the cultural attitudes and values that may impact caregiver experience. As part of a larger, mixed-methods team studying the impact of an innovative psychoeducational intervention, the researchers conducted a qualitative interpretive meta-synthesis (QIMS) to better understand the experiences and perceptions of African Americans who care for family members with ADRD. A QIMS was chosen as the methodology for this study because of its ability to create a more holistic understanding of the phenomenon, while maintaining the integrity of the original studies. An exhaustive literature search yielded 1,285 potentially relevant studies. Studies were compared across a priori inclusion criteria. Findings of this study indicate that overall knowledge of ADRD is relatively low among caregivers and participants are unsure of how to access educational materials. Synthesis of these studies also indicate a need for incorporating spiritual well-being into caregiving services. Results of this study may help social workers and other health care professionals to better understand cultural perceptions of the disease and how to better provide psychoeducational interventions related to the specific needs of African American caregivers.

